# Supplementary material for: Cloud BioLinux: pre-configured and on-demand bioinformatics computing for the genomics community
Source: BMC Bioinformatics. 2012 Mar 19;13:42. doi: 10.1186/1471-2105-13-42 (PMC3372431; doi:10.1186/1471-2105-13-42)
Supplement: Additional file 1 — Supplementary 1 Cloud BioLinux software documentation in the form of a mini, self-contained website. Users need to download and uncompress the .zip file, and open through a web browser the "index.html" file available on the main directory. (ZIP 1823 kb). [file 1471-2105-13-42-S1.ZIP › Cloud-BioLinux-Package-Documentation/docs/pfw.html]

Bio-Linux Software Documentation Pages

Back to search form

## pfw

|  |  |
| --- | --- |
| Name | pfw |
| Description | **pfw** is part of the pftools package, a collection of tools making use of generalised profiles for sequence analysis.  **pfw** computes new weights for individual sequences in a multiple sequence alignment using the method of Sibbald and Argos (1990).  **References:**  Sibbald PR and Argos P. (1990). Weighting aligned protein of nucleic acid sequences to correct for unequal representation. J. Mol. Biol. 216:813-818. |
| Homepage | http://www.isrec.isb-sib.ch/profile/profile.html |
| Remote Documentation | http://www.isrec.isb-sib.ch/ftp-server/pftools/pft2.3/documentation/pfw.1.html |
